# Sex‐associated differences in brain insulin signaling and energy metabolism uncover early pathological alterations driving Alzheimer’s disease neuropathology

**DOI:** 10.1002/alz.085410

**Published:** 2025-01-03

**Authors:** Simona Lanzillotta, Antonella Tramutola, Bindu D Paul, Fabio Di Domenico, Marzia Perluigi, Eugenio Barone

**Affiliations:** ^1^ Sapienza University of Rome, Rome Italy; ^2^ Sapienza University of Rome, Rome, Rome Italy; ^3^ Johns Hopkins University, Baltimore, MD USA

## Abstract

**Background:**

Biological sex influences Alzheimer’s disease (AD) development, particularly concerning brain insulin resistance (bIR) and early energy metabolism defects. Biliverdin reductase‐A (BVR‐A) plays a crucial role in insulin signaling, and its downregulation leads to bIR. However, the sex‐related differences in AD neuropathology and underlying mechanisms remain unclear. We aimed to identify early changes in brain insulin signaling in males and females, shedding light on pathological signs preceding overt bIR and AD neuropathology.

**Method:**

C57BL/6J mice (WT or BVR‐A knock‐out, M and F, n = 10/sex/group) received a chow or a high‐fat diet (HFD, 60% kcal by fat) for 1 or 8 weeks. Peripheral metabolic measurements (fasting glucose, insulin, and OGTT) and cognitive tests (NOR and Y‐maze) were performed. Brain insulin signaling activation (basal and intranasal insulin‐induced), oxidative stress marker levels, mitochondrial activity, and AD neuropathology markers were evaluated in the hippocampus and cortex. Correlations were performed with peripheral metabolic measurements (fasting glucose, insulin, and OGTT) and cognitive tasks (spatial memory).

**Result:**

Male mice exhibited more pronounced alterations in brain insulin signaling, mitochondrial activity, oxidative stress, and AD‐related neuropathology compared to female mice after 8 weeks of HFD. These changes initially manifested in the cortex and subsequently in the hippocampus. Intriguingly, despite these molecular alterations, male mice showed better cognitive performances than female mice following 8 weeks of HFD. This evidence suggests that female mice are more susceptible to these metabolic alterations and that subtle changes promote worse outcomes on cognitive tasks. BVR‐A levels were found decreased in both cortex and hippocampus following HFD, and these changes were sex‐dependent. Loss of BVR‐A significantly correlated with the observed molecular alterations, as confirmed in BVR‐A knock‐out mice. Notably, knock‐out mice exhibited similar alterations observed in C57BL/6J mice after just 1 week on HFD, emphasizing the pivotal contribution of BVR‐A.

**Conclusion:**

This study highlights the importance of identifying sex‐related differences as well as shared features in the progression of bIR in AD. Loss of BVR‐A is a common event, although temporal‐ and regional‐associated differences in males and females are observed. These observations warrant further investigations to unveil early pathological changes driving AD neuropathology.

